# Ultrathin, all-organic, fabric-based ferroelectret loudspeaker for wearable electronics

**DOI:** 10.1016/j.isci.2022.105607

**Published:** 2022-11-17

**Authors:** Moritz Ploner, Ningzhen Wang, Chao Wu, Robert Daniels, Jindong Huo, Gregory A. Sotzing, Yang Cao

**Affiliations:** 1 Electrical Insulation Research Center, Institute of Materials Science, University of Connecticut, Storrs, CT 06269, USA; 2 Department of Chemistry, University of Connecticut, Storrs, CT 06269, USA; 3 Department of Electrical and Computer Engineering, University of Connecticut, Storrs, CT 06269, USA

**Keywords:** Electrical engineering, Polymers, Electronic materials

## Abstract

All-organic, flexible, and body-compatible loudspeakers have become increasingly attractive for wearable electronics. Due to their remarkable piezoelectric response, ferroelectrets are suitable for loudspeakers. Two distinct kinds of ultrathin ferroelectrets, including cellular polypropylene films and expanded polytetrafluoroethylene (ePTFE) films, were combined with three different types of electrodes ((Poly(3,4-ethylenedioxythiophene):poly(styrene sulfonate) (PEDOT:PSS))-coated fabrics, PEDOT:PSS direct coating, and sputter-coated Au/Pd) for study regarding their frequency-dependent sound intensity and radiation directivity. Among the loudspeakers investigated, the all-fabric loudspeakers with ePTFE ferroelectret and PEDOT:PSS-coated spandex electrodes have a higher frequency dependency. Loudspeakers equipped with PEDOT:PSS-coated spandex electrodes are less angle dependent compared to other loudspeakers evaluated. Moreover, the soft loudspeaker constituted of an all-organic FEP(fluorinated-ethylene-propylene)-ePTFE-based ferroelectret and PEDOT:PSS-coated fabrics presented in this paper is easy to integrate with clothes and has a higher thermal stability. It is naturally compatible with the human body and a competitive candidate for future developments of all-organic loudspeakers for wearable electronic systems.

## Highlights

• Ultrathin, all-organic, fabric-based ferroelectret loudspeaker is developed
• The loudspeaker with fabric electrodes shows quasi-omnidirectional acoustic radiation
• The all-organic ePTFE-based loudspeaker works well in the frequency of 5–15 kHz
• This new loudspeaker is compatible with the human body, easy to integrate with clothes

## Graphical abstract

**Figure:**
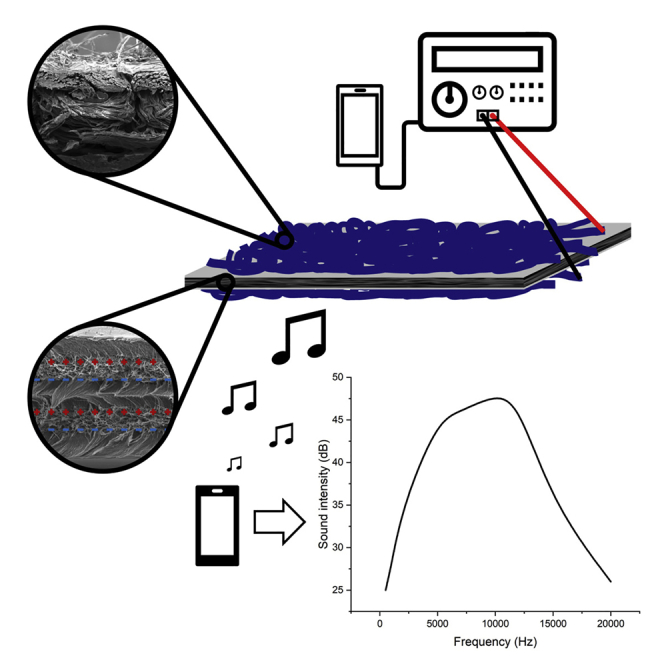

## Introduction

The growing interest in the application of wearable electronics, due to the rapid growth of the emerging Internet of Things (IoT) and body area networks (BANs) for health care monitoring and entertainment such as wearable and portable personal audio systems, has challenged researchers to pursue alternative approaches to manufacture body-compatible, flexible, lightweight, and ultrathin speakers.^1^^,^^2^ Such audio systems can be used for various applications such as integrated helmet headphones, in automobiles and aircrafts,^3^^,^^4^ or as shown in Li et al.^5^ inside a flag replacing conventional speakers. Acoustic capabilities of wearable electronics are becoming essential in the interaction between human and flexible electronics; however, audio systems which contain metal electrodes can no longer meet these requirements. In addition, recent development has shifted from performance-oriented devices to fully wearable electronics with user skin-friendliness. Although fabrics with metal-coated threads (E-textiles) or metallic paints used for coating, e.g., silver nano-ink, are soft and flexible, they could cause allergic reactions and are therefore incompatible with the human body.^6^^,^^7^

Considering the human body compatibility, conductive fabrics offer a possible solution to this problem. Such fabrics, which can be easily inserted into any kind of clothes, are soft, lightweight, breathable, and skin-friendly.^8^ Poly(3,4-ethylenedioxythiophene):poly(styrene sulfonate) (PEDOT:PSS)-coated fabrics seem to be one of the most promising candidates among all conductive polymer-based flexible materials because of its solution processability and high conductivity.^9^^,^^10^

Many diverse types of loudspeakers with various working principles, such as piezoelectric thermoacoustic, and electrostatic technologies,^11^ have been developed for manufacturing flexible, lightweight, and ultrathin wearable speakers. The need for flexible materials cuts out brittle piezoelectric ceramics with high piezoelectric coefficients, such as lead zirconate titanate or zinc oxide. Polyvinylidene fluoride (PVDF) and its copolymers, on the other hand, are highly flexible and easy to process^12^^,^^13^^,^^14^ but exhibit a low piezoelectric coefficient (*d*_33_PVDF ∼30 pC/N). Another relatively new class of functional polymer is ferroelectret materials^15^. Such materials are flexible and have a satisfactory performance due to internal cavities that function as macro-dipoles after internal dielectric-barrier discharges.^16^^,^^17^ Separated trapped charges forming the dipoles yield a piezoelectric coefficient of several hundreds of pC/N. Ferroelectrets show a comparable *d*_33_ value as piezoelectric ceramics but are much more flexible. In comparison to the PVDF and its copolymers, the piezoelectric coefficient of ferroelectrets is much higher.^18^ Cellular polypropylene (PP) is the earliest and most extensively studied ferroelectret. It is commercially available and widely used in flexible nanogenerators, sensors, and actuators.^19^ However, its d_33_ value quickly drops above a temperature of 60°C.^20^ Apart from their low temperature resistance, PP films show a closed structure which limits the charge density.

Materials with better temperature resistance and higher charge densities are desired for better stability and performance. Expanded polytetrafluoroethylene (ePTFE) ferroelectret films containing porous or tubular channel structures are much more temperature resistant and have an open porous structure which enables prefilling of ions and the deployment of much more charges during corona poling, leading to a higher charge density and a higher electric potential and could be a satisfactory solution for high-temperature-resistant ferroelectret loudspeakers.^21^^,^^22^^,^^23^ FEP(fluorinated-ethylene-propylene)-ePTFE-films with a higher number of layers lead to a better performance due to a higher number of macro-dipoles (the five-layered film FEP-ePTFE-FEP-ePTFE-FEP has two macro-dipoles).^18^

In this paper, two diverse kinds of ferroelectrets combined with three different types of electrodes have been investigated regarding their sound intensity over a frequency range from 100 Hz to 20 kHz. A five-layered ePTFE-FEP ferroelectret film (the FEP-ePTFE-FEP-ePTFE-FEP), which is a good tradeoff between number of macro-dipoles and elastic modulus, is combined with PEDOT: PSS-coated fabrics to form an all-organic, wearable loudspeaker, which is flexible, can be easily worn, and is less allergic.

### Experiments

#### Fabrication of different paper-thin loudspeakers and ferroelectret nanogenerators

**Figure 1:**
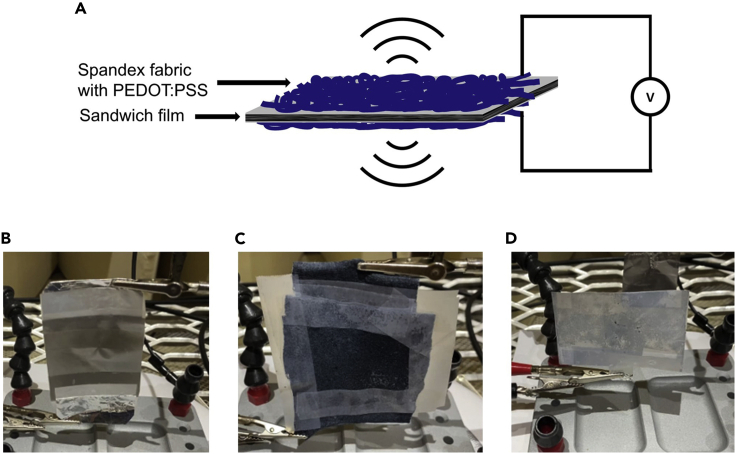
Loudspeaker fabrication (A) Schematic diagram of the working principle of a ferroelectret loudspeaker. (B) Sputter-coated polypropylene ferroelectret loudspeaker. (C) FEP-ePTFE-FEP-ePTFE-FEP ferroelectret with PEDOT:PSS-coated fabric loudspeaker. (D) PEDOT:PSS-coated polypropylene ferroelectret loudspeaker.

The schematic of the ferroelectret loudspeaker is shown in Figure 1A. The sputter-coated polypropylene ferroelectret loudspeaker is displayed in Figure 1B. The FEP-ePTFE-FEP-ePTFE-FEP ferroelectret and the PP film respectively have been attached to the PEDOT:PSS-coated fabrics using tape, as shown in Figure 1C. The connection to the sound amplifier (Gemini^TM^ XG-2000) output has been established using the conductive fabrics itself. The PEDOT:PSS-coated polypropylene ferroelectret loudspeaker, as displayed in Figure 1D, has been prepared with electrodes already attached to the film. The connections to the sound amplifier have been established using aluminum foil which has been attached to the electrodes using tape.

To evaluate the piezoelectric performance of the PP and ePTFE-based ferroelectrets, both materials have been prepared as ferroelectret nanogenerators after covering with PEDOT:PSS fabric electrodes.^18^ The electroactive area of the nanogenerators is 13 × 13 mm^2^. A cyclic compressive force of 30 N with a frequency of 1 Hz was applied and the piezoelectric responses including current and voltage were recorded using a Keithley 6514 meter.

#### Sound waveform generation and recording

**Figure 2:**
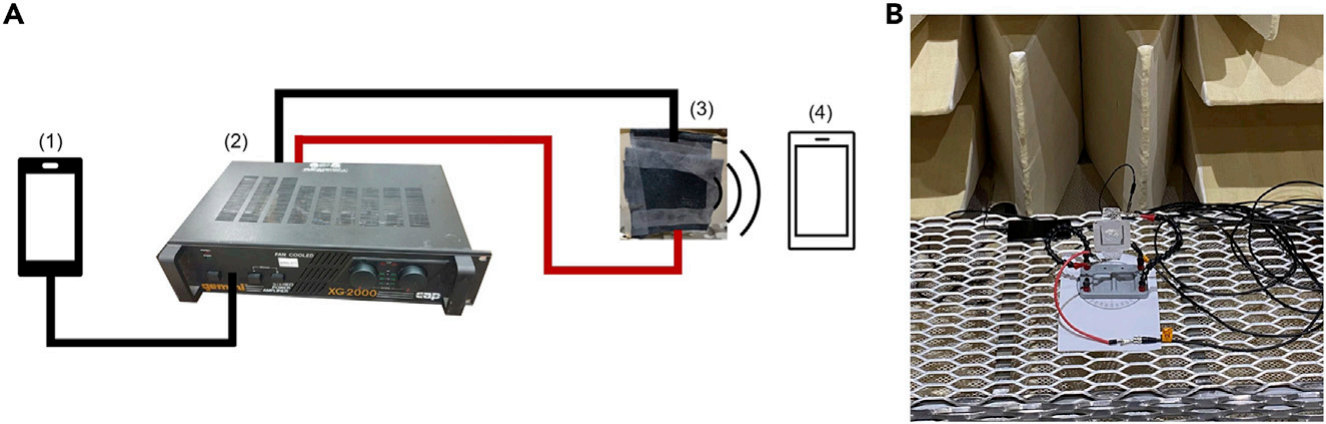
Test setup (A) Test setup used for sound intensity tests of different ferroelectret loudspeakers. (B) Test setup inside the anechoic chamber.

A cellphone (item 1 in Figure 2) was used to generate sinusoidal signals with a specific frequency and connected to the sound amplifier (item 2). The output ports of the sound amplifier were connected to the different loudspeakers one at a time for testing (item 3). The voltage applied to the different loudspeaker configurations, which corresponds to the volume output at the amplifier, was kept the same for all samples. The sound intensity was measured using a second cellphone (item 4). The smartphone app “Physics Toolbox Sensor Suite” by Vieyra Software has been used for sound measurement and recording (Sound Meter, Tone Detection, Tone Generation, Spectrum Analyzer). The measurement has been conducted at the exact same position (center of the active area of the loudspeaker) and the same distance from the active area (5 cm) for all the types of loudspeakers investigated to guarantee comparable results. The entire setup, except the sound amplifier, was placed inside an anechoic chamber for the experiments.

## Results and discussion

The working principle of a ferroelectret speaker is based on charges trapped inside the internal cavities (such as foam cells, pores, etc.) of the ferroelectret membrane. Trapped and separated charges are the result of internal dielectric-barrier discharges which are initiated by high electric fields. Those macro-dipoles respond to externally applied fields and either compress or expand, depending on the polarity of the external field. Other than the function of a nanogenerator,^18^ which leads to the formation of an electric field if mechanical deformation is applied, a loudspeaker reacts to the external field by means of expanding or compression. The electric field reshapes the macro-dipoles inside the ferroelectret and depending on the magnitude of the applied voltage, more dipoles contract or expand. Such mechanical deformation is the same principle as the vibration of a membrane of usual loudspeakers and leads to the emission of soundwaves, as shown in Figure 1A.

### Electrode size and layout

**Figure 3:**
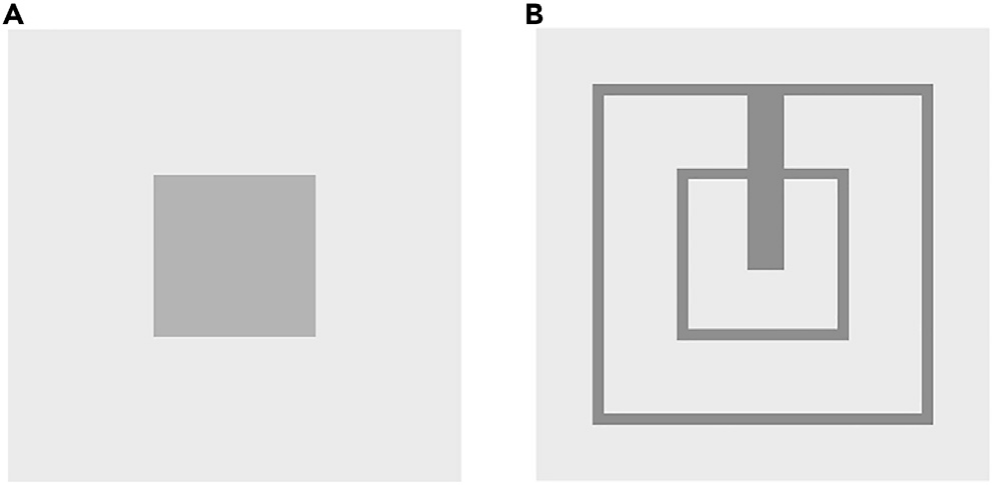
Electrodes layout (A) Concentrated electrode on polypropylene ferroelectret. (B) Distributed electrode on polypropylene ferroelectret.

To evaluate the impact of the size of the electrode on the sound intensity of the speaker, different samples of the sputter-coated polypropylene ferroelectret loudspeaker with electrode areas of 26 cm^2^, 13.5 cm^2^, 8 cm^2^, and 1.5 cm^2^, respectively, have been prepared. Furthermore, to investigate the impact of the layout of the electrode, two different electrode shapes of the size of 8 cm^2^ have been deployed on the PP ferroelectret loudspeaker, as displayed in Figure 3. The sound intensity has been recorded for a frequency range from 100 Hz to 20 kHz.

**Figure 4:**
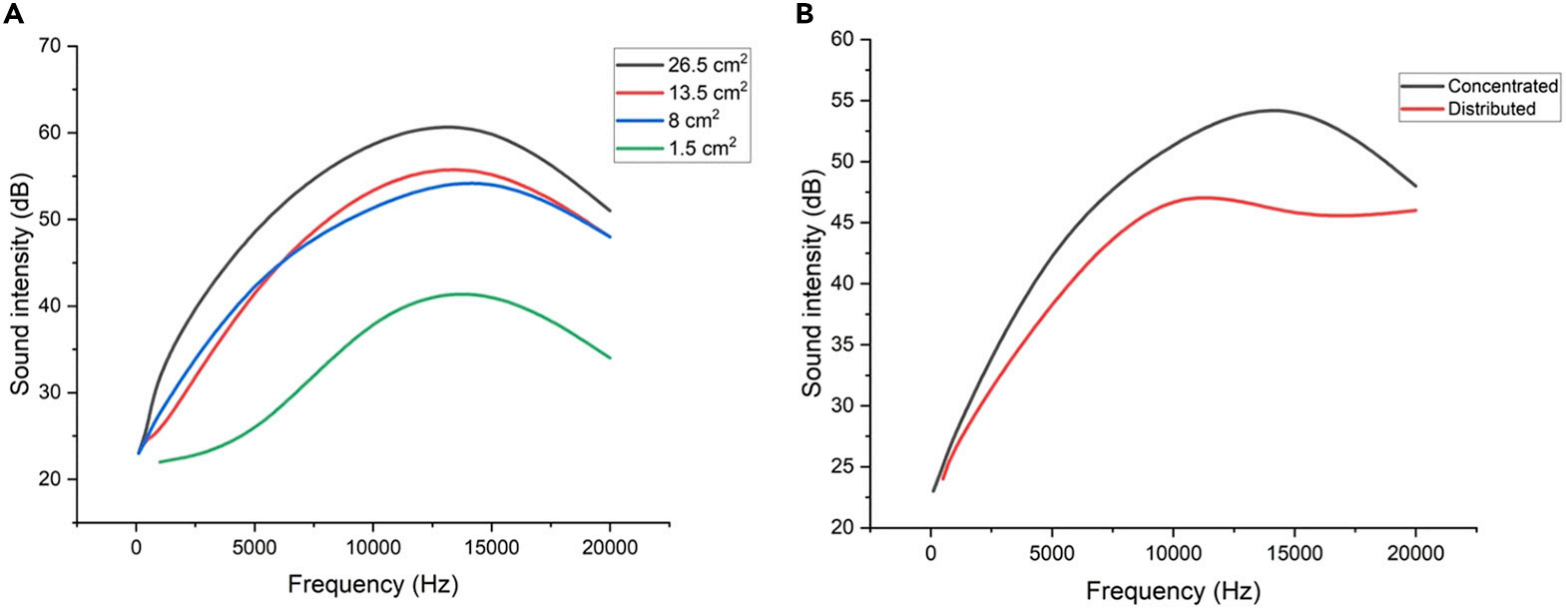
Sound intensity measurements (A) Sound intensity over a frequency range of 100 Hz–20 kHz for the sputter-coated PP ferroelectret loudspeakers with different electrode sizes. (B) Sound intensity over a frequency range of 100 Hz to 20 kHz for the sputter-coated polypropylene ferroelectret loudspeakers with different electrode layouts.

The results in Figure 4A show that a larger conductive area results in a higher sound intensity since a larger number of macro-dipoles expand and compress as reaction to the external voltage due to the distribution of the electric field over a larger area, leading to a more intense sound wave. The size does not appear to change the frequency response of sputter-coated PP ferroelectret loudspeakers in the frequency range of 10 to 15 kHz significantly. The different shape of the curve for a size of 1.5 cm^2^ should be due to measurement errors of the low sound intensity in relative to noise.

Figure 4B shows a more concentrated conductive area leads to a more intense sound wave. A more distributed electrode leads to a flatter sound intensity in the frequency range of 10 to 20 k Hz. However, not only the magnitude of the sound intensity but also the frequency dependence of the intensity varies, which could be related to the different acoustic vibration modes caused by the layout of the electrodes. Video S1 shows the recording of the sputter-coated PP ferroelectret loudspeaker playing the song “UConn Husky”.

Video S1. Recording of the sputter coated PP ferroelectret loudspeaker playing the song “UConn Husky”

### Electrode type

To study the respective influences of different electrode materials, different ferroelectret-based loudspeakers with varying electrode types have been fabricated and assessed. The different loudspeakers have the same size of active material (ferroelectret) and the same-sized electrodes (33 cm^2^), enabling comparison between the different samples. The sound intensity for different materials over the frequency range of 100 Hz to 20 kHz has been recorded.

**Figure 5:**
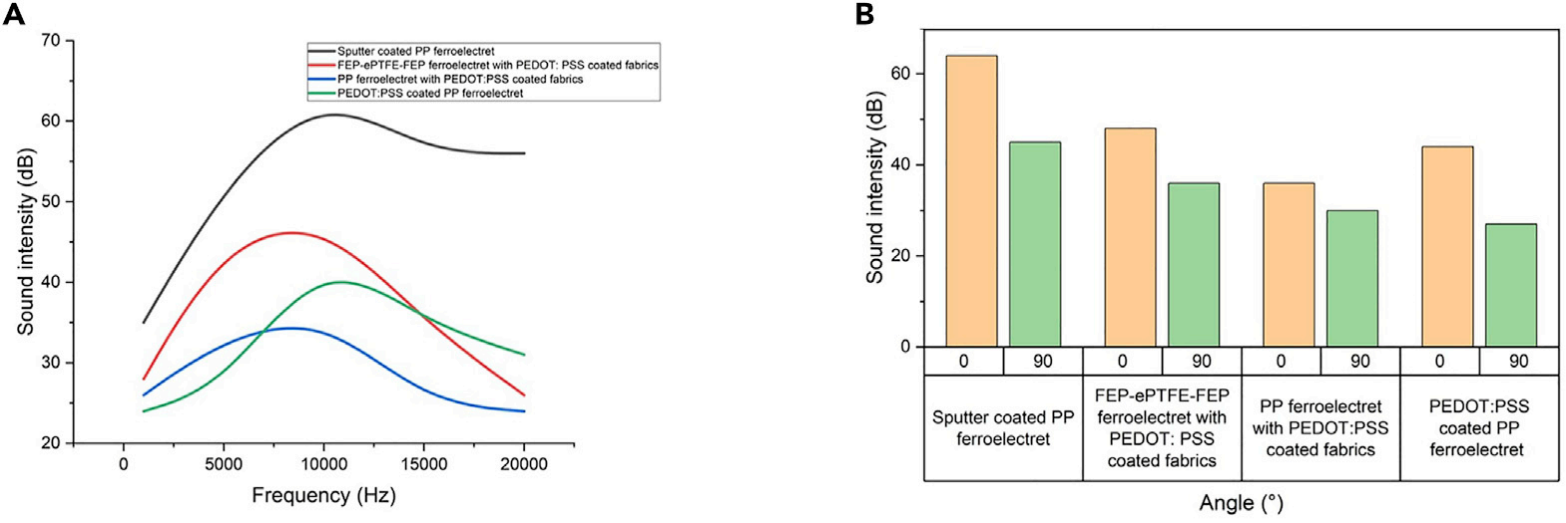
Sound intensity measurements with different electrodes (A) Sound intensity versus frequency for different electrode materials. (B) Sound intensity versus measurement angle for different electrode materials.

The results in Figure 5A show a higher sound intensity for loudspeakers electrode with PEDOT: PSS-coated fabrics compared to direct coating with PEDOT:PSS (e.g., PP EMFit films). Furthermore, direct coating of PEDOT:PSS on PP films is not very stable and easily wears off, and the recording of this loudspeaker playing the song “UConn Husky” is shown by Video S2. More importantly, fabrics are easier to coat, prepare, and to insert in clothes compared to the direct-coated PP films.

Video S2. Recording of the direct coated PEDOT:PSS on PP ferroelectret loudspeaker playing the song “UConn Husky”

**Figure 6:**
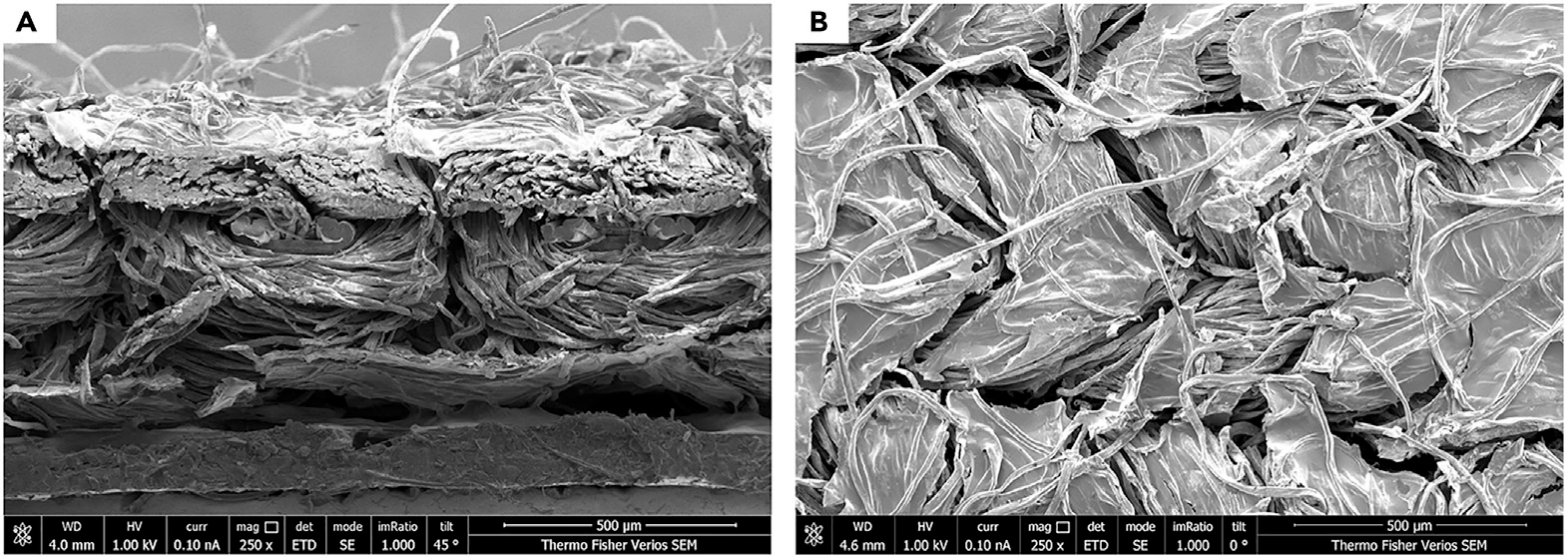
SEM images of conductive fabrics (A) SEM image of side view of PEDOT:PSS-coated fabrics (90% cotton, 10% spandex). (B) SEM image of top view of PEDOT:PSS-coated fabrics (90% cotton, 10% spandex).

The results furthermore underline the high frequency dependency of PEDOT: PSS-coated fabrics, especially when the frequency is higher than 7.5 kHz. For both samples, namely the FEP-ePTFE-FEP-ePTFE-FEP ferroelectret with PEDOT: PSS-coated fabrics and the polypropylene ferroelectret with PEDOT:PSS-coated fabrics, the sound intensity decreases for higher frequency values, while the direct-coated PP film and the sputter-coated PP film show a more saturated sound intensity also for higher frequencies, as shown in^11^ as well. The PEDOT:PSS-coated fabrics show a highly frequency-dependent response. Due to the porous structure of the fabrics, as seen in the SEM image displayed in Figure 6, the fabric electrodes could be considered as damping layers, and waves of specific frequency ranges can be damped. For resonant frequency range on the other hand, the whole configuration of fabric and ferroelectret responds to the voltage applied and vibrates.

Comparing to the results for the polypropylene ferroelectret with PEDOT:PSS-coated fabrics loudspeaker, the sputter-coated polypropylene ferroelectret loudspeaker shows a much higher sound intensity. The reason for the worse performance is the lower conductivity of the PEDOT: PSS-coated fabrics compared to the sputtered electrode. The sheet conductivity of the fabric electrodes is 0.026 S/cm. Additionally, the electrode might not be fully attached due to the process of how the speaker was assembled. As seen in the previous chapter, the electrode size is key regarding the sound intensity. A not fully attaching electrode corresponds to a smaller sized effective electrode and therefore yields less sound intensity.

To validate the directivity of the loudspeaker, the sound intensity has been measured at different angles relative to the loudspeaker. An angle of 0° corresponds to the direct facing of the recording device with the biggest active area of the loudspeaker. 90° means the alignment of the recording device and the loudspeaker so that the active area of the loudspeaker does not directly face the recording device.

The results displayed in Figure 5B show a lower angle dependency for loudspeakers with the PEDOT: PSS-coated fabrics electrodes than the same types of loudspeakers with the other electrode materials assessed. For speakers with PEDOT: PSS-coated fabrics electrodes, they still work with a remarkable sound intensity at an angle of 90°, as displayed in Table 1. As mentioned in Wang et al.^18^, the PEDOT: PSS-coated fabrics form an additional macro-dipole and lead to the expansion and compression of the fabric if voltage is applied, contributing to the overall piezoelectric performance of the loudspeaker. The vibration of the fabrics itself leads to a better sound distribution in different directions. Moreover, this quasi-omnidirectional acoustic radiation for loudspeaker with PEDOT: PSS fabrics electrodes could be attributed to the vibrational damping characteristics of the fabrics while sufficiently high sound quality was achieved.

**Table 1:** Percentage of reduction of sound intensity for an angle of 90° relative to the loudspeaker

	Sputter-coated PP ferroelectret	FEP-ePTFE-FEP-ePTFE-FEP ferroelectret with PEDOT: PSS-coated fabrics	PP ferroelectret with PEDOT:PSS-coated fabrics	PEDOT:PSS-coated PP ferroelectret
Reduction (%)	37.5	18.75	16.67	38.63

The PEDOT: PSS-coated fabrics work well in a frequency range of 5000 Hz–12 kHz. Compared to the non-organic sputter-coated PP film, the sound intensity is lower; however, it demonstrates other advantages such as skin-friendliness and biocompatibility, since it is all-organic by nature. Furthermore, it can easily be inserted in clothes or integrated into emerging haptic smart skin, because it is less allergic and more biofriendly.

### Ferroelectret type

**Figure 7:**
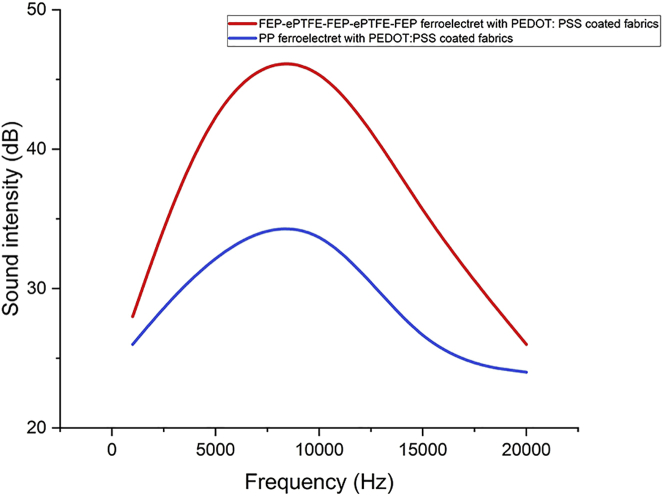
Sound intensity versus frequency for an FEP-ePTFE-FEP-ePTFE-FEP ferroelectret with PEDOT: PSS-coated fabrics and for a polypropylene ferroelectret with PEDOT:PSS-coated fabrics

To determine the respective influence of different ferroelectret materials, varied materials have been used and evaluated. The results shown in Figure 5A demonstrate better performance, being equipped with the same electrode, namely the PEDOT: PSS-coated fabrics, of FEP-ePTFE-FEP-ePTFE-FEP compared to the polypropylene film. The recording of the song “UConn Husky” played by the FEP-ePTFE-FEP-ePTFE-FEP ferroelectret loudspeaker can be found in Video S3. Figure 7 shows the comparison of the sound intensity over the frequency for the two ferroelectret loudspeakers.

Video S3. Recording of the FEP-ePTFE-FEP-ePTFE-FEP ferroelectret loudspeaker with fabric electrodes playing the song “UConn Husky”

**Figure 8:**
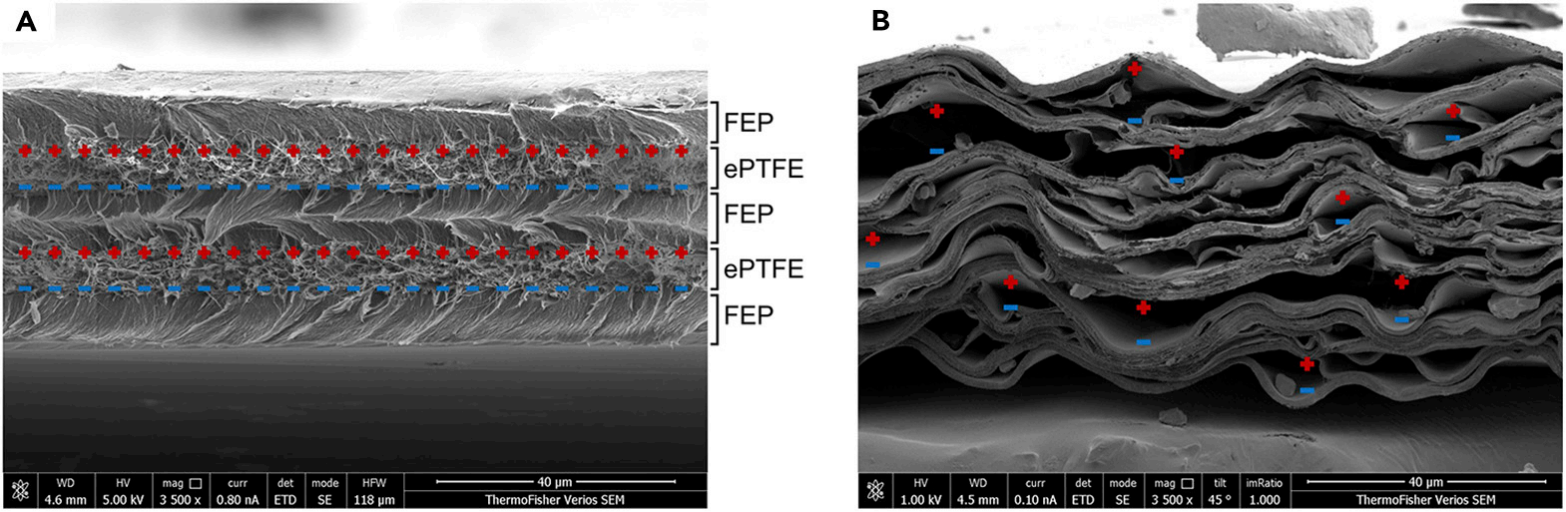
SEM images of ferroelectrets (A) SEM image of a five-layered ePTFE-based ferroelectret film (FEP-ePTFE-FEP-ePTFE-FEP). (B) SEM image of a polypropylene ferroelectret film.

In both ferroelectrets, charges are generated and deployed using internal dielectric-barrier discharges. As displayed in Figure 8, there are two macro-dipoles for the ePTFE-based ferroelctret film and there are several small macro-dipoles inside the PP ferroelectret film. The reason for the higher sound intensity of the FEP-ePTFE-FEP-ePTFE-FEP ferroelectret compared to the PP ferroelectret is its higher piezoelectric performance at this frequency range of mechanical vibration.

**Figure 9:**
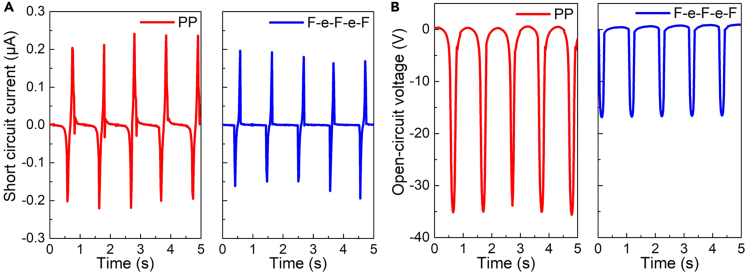
Performance of different ferroelectret nanogenerators (A) Comparison of the short-circuit and (B) open-circuit voltage of the ferroelectret nanogenerators fabricated with PP and FEP-ePTFE-FEP-ePTFE-FEP (abbreviated as F-e-F-e-F) ferroelectret films.

As shown in Figure 9, when assembling the two ferroelectrets as piezoelectric nanogenerators and applying the cyclic force of 30 N at the frequency 1 Hz, the obtained short-circuit current and open-circuit voltage of the ePTFE-based ferroelectret nanogenerator are both lower than that of PP ferroelectret, especially for the voltage. However, when measuring the piezoelectric coefficient *d*_33_ at the frequency of 110 Hz, the *d*_33_ value for polypropylene is 90 pC/N while for an ePTFE-based ferroelectret the *d*_33_ value is 160 pC/N. This means the ePTFE-based ferroelectret has a better performance at a higher frequency, and is more suitable to be utilized as loudspeakers, biosensors, etc. Moreover, the FEP-ePTFE-FEP-ePTFE-FEP ferroelectret shows good charge stability at a higher temperature,^22^ and it was charged one year ago using corona poling, which implies the ePTFE-based ferroelectret is quite stable and an excellent option for wearable loudspeakers.

### Conclusions

Ultrathin, all-organic, fabric-based ferroelectret loudspeakers have been developed, prototyped, and tested in this study. The electrode size plays a key factor for the performance of ferroelectret loudspeaker. Moreover, a more concentrated electrode shows a higher sound intensity compared to a more distributed electrode. However, this must be evaluated to a higher extent in the future.

The PEDOT: PSS-coated fabrics as electrodes show a satisfactory performance and are more frequency selective compared to the other loudspeakers investigated. In addition, loudspeakers with the PEDOT: PSS-coated fabrics as electrodes show beneficial quasi-omnidirectional acoustic radiation possibly due to the damping characteristics. Furthermore, it is skin-friendliness and biocompatibility since it is all-organic nature and can easily be inserted in clothes.

The all-organic paper-thin ePTFE-based ferroelectret works well in the frequency range of 5–15 kHz because of its high *d*_33_ value for higher frequencies and is less angle dependent compared to the other loudspeakers evaluated. Moreover, an even higher charge density and better sound performance can be achieved with prefilling the porosities and cavities of ePTFE-FEP films with ions.

The all-organic FEP-ePTFE- FEP-ePTFE-FEP ferroelectret with PEDOT: PSS-coated fabrics presented is naturally compatible with the human body, easy to integrate with clothes, and can withstand higher temperatures due to their inherent charge stability and are considered competitive candidates for future developments of all-organic loudspeakers for wearable electronics that serve as human-machine interface.

## STAR★Methods

### Key resources table

**Table:** 

REAGENT or RESOURCE	SOURCE	IDENTIFIER
**Chemicals, peptides, and recombinant proteins**		

FEP film	Goodfellow Corp.	FP341013
Expanded PTFE	BHA Altair LLC.	Average pore size 3 μm
Polypropylene ferroelectret	EMFit Ltd.	Thickness 60 μm (+/− 5 μm)
fabric	Nike	90% cotton and 10% spandex
Clevios PH1000	Heraeus	Solids content 1/1.3
DMSO	Sigma-Aldrich	ACS reagent >99.9%

### Resource availability

#### Lead contact

Further information and requests for resources can be provided by the lead contact, Yang Cao (yang.cao@uconn.edu).

#### Materials availability

This study did not generate new unique reagents.

### Experimental model and subject details

This work did not need any unique experimental model.

### Method details

#### Preparation of the FEP-ePTFE-FEP-ePTFE-FEP ferroelectret

The expanded PTFE, with an average pore size of 3 μm, for the FEP-ePTFE-FEP-ePTFE-FEP film, was supplied by BHA Altair, LLC. The FEP film with a thickness of 12.5 μm was supplied by Goodfellow Corp. The five-layered films were laminated together at 285°C for 1 h using a Carver Laboratory hot press. After the lamination, the five-layered film was corona poled with a voltage of +20 kV for 5 min. During corona poling, the charges deposited on the surface of the top layer of the FEP-ePTFE-FEP-ePTFE-FEP film generates high enough electric fields for triggering internal dielectric-barrier discharges (DBDs) inside the porous ePTFE films. The air inside the ePTFE pores was ionized due to Paschen breakdown, resulting in charges separation and trapping of charges of opposite polarities at the interfaces between the ePTFE and FEP films. Those trapped charges form stable macro-dipoles across the pores, which, with increasing numbers form an internal electric field opposite the external field. This leads to the reduction of the total electric field inside the ePTFE and the extinction of the micro-plasma discharges (DBDs) that saturates the dipole polarization.

The *d*_33_ coefficient of the ePTFE-based laminated structures was measured using a PM3500 meter from KCF Technologies, with an applied force of 0.25 N and a frequency of 110 Hz.

#### Preparation of the electrodes

##### Preparation of all-organic polymer-on-fabric electrodes

A dispersion consisting of 95 weight% Clevios PH1000 and five weight% DMSO, provided by Heraeus and Sigma-Aldrich, respectively, was drop-cast onto a fabric (90% cotton, 10% spandex), supplied by Nike, until saturation. The samples were hung to dry at 110°C for 1 h.

##### Preparation of the PEDOT:PSS coated polypropylene ferroelectret

The polypropylene (PP) ferroelectret film was supplied by EMFit Ltd. First, the surface of the PP ferroelectret was cleaned by plasma treatment. The PEDTO:PSS solution was then applied by spin coating and hung to dry.

##### Preparation of the sputter-coated polypropylene ferroelectret

The polypropylene (PP) ferroelectret from EMFit Ltd was sputter coated with 60% gold and 40% platinum for 90 s.

### Additional information

Supplementary Information accompanies this paper.

## Data Availability

All data are available in the paper and in supplemental files, and/or from the corresponding authors upon reasonable request. This paper does not report original code.

## References

[1] Niu S., Matsuhisa N., Beker L., Li J., Wang S., Wang J., Jiang Y., Yan X., Yun Y., Burnett W. (2019). Nat. Electron..

[2] Dsouza H., Schyndel A.v., Pastrana J., Cao Y., Hunter E., Rakerd B., Sepulveda N. (2020). J. Sound Vib..

[3] Guigou C., Fuller C. (1999). J. Sound Vib..

[4] Chen Y.-C., Ko W.-C., Chen H.-L., Wu W.-J., Chang P.-Z., Lee C.-K. (2012). A thin light flexible electromechanically actuated electret-based loudspeaker for automotive applications. IEEE Trans. Ind. Electron..

[5] Li W., Torres D., Díaz R., Wang Z., Wu C., Wang C., Lin Wang Z., Sepúlveda N. (2017). Nanogenerator-based dual-functional and self-powered thin patch loudspeaker or microphone for flexible electronics. Nat. Commun..

[6] Li Z., Sinha S.K., Treich G.M., Wang Y., Yang Q., Deshmukh A.A., Sotzing G.A., Cao Y. (2020). All-organic flexible fabric antenna for wearable electronics. *J. Mater. Chem*. C.

[7] Hostýnek J.J., Hinz R.S., Lorence C.R., Price M., Guy R.H. (1993). Crit. Rev. Toxicol..

[8] Takamatsu S., Lonjaret T., Crisp D., Badier J.M., Malliaras G.G., Ismailova E. (2015). Direct patterning of organic conductors on knitted textiles for long-term electrocardiography.. Sci. Rep..

[9] Wang Y., Zhu C., Pfattner R., Yan H., Jin L., Chen S., Molina-Lopez F., Lissel F., Liu J., Rabiah N.I. (2017). A highly stretchable, transparent, and conductive polymer. Sci. Adv..

[10] Zeng W., Shu L., Li Q., Chen S., Wang F., Tao X.M. (2014). Fiber-based wearable electronics: a review of materials, fabrication, devices, and applications. Adv. Mater..

[11] Yildirim A., Grant J.C., Song G., Yook S., Mutlu Z., Peana S., Dhanabal A., Sinha S.K., Daniels R., Bellisario K.M. (2020). Roll-to-Roll production of novel large-area piezoelectric films for transparent, flexible, and wearable fabric loudspeakers. Adv. Mater. Technol..

[12] Hübler A.C., Bellmann M., Schmidt G.C., Zimmermann S., Gerlach A., Haentjes C. (2012). Fully mass printed loudspeakers on paper. Org. Electron..

[13] Sugimoto T., Ono K., Ando A., Kurozumi K., Hara A., Morita Y., Miura A. (2009). PVDF-driven flexible and transparent loudspeaker. Appl. Acoust..

[14] Lu L., Ding W., Liu J., Yang B. (2020). Flexible PVDF based piezoelectric nanogenerators. Nano Energy.

[15] Zhang Y., Bowen C.R., Ghosh S.K., Mandal D., Khanbareh H., Arafa M., Wan C. (2019). Ferroelectret materials and devices for energy harvesting applications. Nano Energy.

[16] Qiu X., Carpi F. (2016). Electromechanically Active Polymers. A Concise Reference.

[17] Qiu X., Mellinger A., Wegener M., Wirges W., Gerhard R. (2007). J. Appl. Phys..

[18] Wang N., Daniels R., Connelly L., Sotzing M., Wu C., Gerhard R., Sotzing G.A., Cao Y. (2021). All-organic flexible ferroelectret nanogenerator with fabric-based electrodes for self-powered body area Networks. Small.

[19] Li W., Torres D., Wang T., Wang C., Sepúlveda N. (2016). Flexible and biocompatible polypropylene ferroelectret nanogenerator (FENG): on the path toward wearable devices powered by human motion. Nano Energy.

[20] Wu N., Cheng X., Zhong Q., Zhong J., Li W., Wang B., Hu B., Zhou J. (2015). Cellular polypropylene piezoelectret for human body energy harvesting and health monitoring. Adv. Funct. Mater..

[21] Altafim R.A.P., Qiu X., Wirges W., Gerhard R., Altafim R.A.C., Basso H.C., Jenninger W., Wagner J. (2009). Template-based fluoroethylenepropylene piezoelectrets with tubular channels for transducer applications. J. Appl. Phys..

[22] Xia Z., Gerhard-Multhaupt R., Künstler W., Wedel A., Danz R. (1999). High surface-charge stability of porous polytetrafluoroethylene electret films at room and elevated temperatures. J. Phys. D Appl. Phys..

[23] Zhukov S., Fedosov S., von Seggern H. (2011). Piezoelectrets from sandwiched porous polytetrafluoroethylene (ePTFE) films: influence of porosity and geometry on charging properties. J. Phys. D Appl. Phys..

